# Ion Channel-Targeting Modulators in Glioma: Pharmacological Advances and Therapeutic Perspectives

**DOI:** 10.3390/ijms27188167

**Published:** 2026-09-14

**Authors:** Sheng-Nan Wu, Sheng-Che Lin, Rasa Liutkevičienė

**Affiliations:** 1 Department of Medical Research, An Nan Hospital, China Medical University, No. 66, Section 2, Changhe Road, An Nan District, Tainan 70965, Taiwan; 2 Division of Plastic Surgery, Department of Surgery, An Nan Hospital, China Medical University, Tainan 70965, Taiwan; 3 Laboratory of Ophthalmology, Institute of Neuroscience, Lithuanian University of Health Sciences, Eivenių St. 4, LT-50161 Kaunas, Lithuania; rasa.liutkeviciene@lsmuni.lt

**Keywords:** glioma, ion channel, channel kinetics, ionic current, small molecules, docking prediction

## Abstract

Highly aggressive gliomas remain difficult to treat because of their marked invasiveness, frequent recurrence, and association with glioma-related epilepsy. Increasing evidence suggests that ion channels expressed in glioma cells, including voltage-gated Na^+^ channels, large-conductance Ca^2+^-activated K^+^ (BK_Ca_) channels, intermediate-conductance Ca^2+^-activated K^+^ (IK_Ca_) channels, and inwardly rectifying K^+^ (Kir) channels, contribute to these malignant phenotypes and represent potential therapeutic targets. This perspective summarizes current evidence for eight representative drugs and phytoconstituents—perampanel, valproic acid, cilostazol, berberine, oxaliplatin, temozolomide, arecoline, and triptolide—that modulate these ion channels. Perampanel and valproic acid suppress voltage-gated Na^+^ current in glioma cells and may provide additional benefits in controlling glioma-associated epilepsy. Cilostazol activates BK_Ca_ channels, whereas berberine, oxaliplatin, and temozolomide inhibit IK_Ca_ channel activity. Arecoline suppresses IK_Ca_ and Kir channel activity, while triptolide inhibits Kir channels. These observations suggest that ion channel modulation may influence glioma cell proliferation, migration, invasion, and excitability. To complement the available experimental evidence, we performed molecular docking analyses as a hypothesis-generating approach to explore potential ligand–channel interactions. Because these computational predictions have not been experimentally validated, they should be interpreted cautiously and viewed as a framework for future mechanistic studies rather than definitive evidence of binding. We also discuss important translational considerations, including the limited clinical evidence and the toxicity profiles of several compounds. In particular, arecoline is associated with carcinogenicity and neurotoxicity, triptolide with significant systemic toxicity, berberine with poor oral bioavailability and potential drug interactions, and oxaliplatin with dose-limiting neurotoxicity. Overall, this perspective highlights ion channels as promising therapeutic targets in glioma while emphasizing the need for rigorous experimental validation and careful evaluation of safety before clinical translation.

## 1. Introduction

Glioma, arising from non-neuronal glial cells within the central nervous system, presents one of the most formidable challenges in modern neuro-oncology. Among these, glioblastoma multiforme represents a highly malignant phenotype characterized by rapid progression, extensive tissue invasiveness, and an exceptionally poor clinical prognosis [1,2]. Despite substantial iterative advances in neurosurgical techniques, radiotherapy, and conventional chemotherapy, patient life expectancy remains severely constrained, underscoring the urgent need for novel, mechanism-driven therapeutic strategies.

Accumulating evidence highlights a pivotal pathophysiological framework: glioma cells actively exploit endogenous ion channels to drive their malignant behaviors. Four principal ion channel classes are functionally expressed on the glioma plasma membrane and orchestrate key aspects of tumor progression: voltage-gated Na^+^ (Na_V_) channels, large-conductance Ca^2+^-activated K^+^ (BK_Ca_) channels, intermediate-conductance Ca^2+^-activated K^+^ (IK_Ca_) channels, and the inwardly rectifying K^+^ (Kir) channels [2,3,4,5].

Rather than acting merely as passive electrical conduits, these channels serve as dynamic upstream regulators that modulate intracellular signaling cascades, facilitate the rapid volume changes necessary for active migration/invasion, and promote resistance to programmed cell death [3,4,5]. Consequently, targeting the biophysical properties of these channels offers an attractive, yet largely unexplored, therapeutic vulnerability.

In this perspective, we provide a critical assessment of the therapeutic implications of targeting ion channels in glioma, with particular emphasis on eight representative bioactive compounds selected from both clinically established pharmaceuticals and experimental phytoconstituents (Table 1). The compounds were selected based on one or more of the following considerations: (i) evidence of ion channel modulation relevant to glioma biology, (ii) regulatory approval for clinical use (e.g., drugs approved by the U.S. Food and Drug Administration [FDA] or the European Medicines Agency [EMA], or drugs that have reached advanced clinical development), (iii) potential for drug repurposing in glioma therapy, (iv) availability of experimental evidence, including electrophysiological, cellular, and/or preclinical studies supporting their mechanisms of action, and (v) translational relevance and therapeutic potential. It must be emphasized that representative natural compounds with well-documented ion channel-modulating activities and preclinical evidence in glioma were included to provide a broader perspective on emerging therapeutic strategies. This work highlights recent findings and emerging mechanistic insights, including the functional consequences of ion channel modulation on glioma cell proliferation, migration, invasion, the tumor microenvironment, and therapeutic response. Given the growing clinical limitations of single-target agents, exploring how these diverse structures modulate specific channel gates provides a rational foundation for innovative drug repurposing and multi-target therapeutic designs [6,7,8,9].

To complement this synthesis and stimulate future experimental validation, we introduce original atomic-level molecular docking simulations for selected compounds. It is important to emphasize that these computational models are presented here not as definitive mechanistic proof, but strictly as a complementary, hypothesis-generating tool to visualize plausible ligand–channel binding modalities, hydrogen bonds, and hydrophobic contacts where experimental crystal structures remain unavailable. To ensure full methodological transparency and reproducibility, the detailed docking protocols are provided in the Appendix A. Ultimately, this perspective aims to bridge the gap between electrophysiological observations and structural predictions, offering fresh insights into how targeted ion channel modulation might improve clinical outcomes in glioma therapy.

## 2. Voltage-Gated Na^+^ (Na_V_) Channel

The voltage-gated Na^+^ channels, known as Na_V_ channels, play a critical role in initiating and propagating action potentials in excitable cells. In mammals, nine distinct Na_V_ channel α-subunits (Na_V_1.1–1.9) are expressed across various excitable tissues. The α-subunits of these channels have homologous domains (DI–DIV), each with six transmembrane segments (S1–S6). They are thought to be analogous to the four separate subunits of the voltage-gated K^+^ channel, which contain a pore domain formed by S1–S4 segments located toward the periphery of the channel [21,22]. During the rapid depolarization phase of the action potential, Na_V_ channels produce voltage-gated Na^+^ currents (*I*_Na_) instantaneously. A brief depolarization stimulus rapidly activates *I*_Na_ within a few milliseconds, after which the current undergoes time-dependent inactivation over the subsequent tens of milliseconds [21,22,23]. *I*_Na_ is readily elicited by depolarizing shifts in membrane potential that are detected by the channel’s voltage-sensing domains, which are tightly coupled to the pore-forming domain and mediate channel opening [21,22,23]. The non-inactivating (persistent) *I*_Na_, elicited by prolonged ramp pulses, has also been identified as a key contributor to membrane excitability in glioma cells [24]. Increasing evidence has revealed that the expression of Na_V_ channels extends beyond electrically excitable cells. Notably, many cancer cell types express functional Na_V_ channels, despite being incapable of generating action potentials, highlighting noncanonical roles for these channels in tumor biology [10,24,25,26,27].

### 2.1. Perampanel

Perampanel (2-(2-oxo-1-phenyl-5-pyridin-2-yl-1,2-dihydropyridin-3-yl)benzonitrile, 3-(2-cyanophenyl)-5-(2-pyridyl)-1-phenyl-1,2-dihydropyridin-2-one, E2007, Fycompa^®^) is the first approved antiepileptic drug in the class of selective non-competitive antagonists of α-amino-3-hydroxy-5-methyl-4-isoxazolepropionic acid (AMPA)-type receptors [28]. This class of AMPA-type receptors represents the predominant subtype of ionotropic glutamate receptors. Perampanel has been demonstrated to reduce neuronal excitation mainly through the blockade of AMPA receptors [28]. As a once-daily oral antiepileptic drug, it has been approved globally for the adjunctive treatment of partial epilepsy and primary generalized tonic–clonic seizures [28,29], although the underlying mechanism of action through which perampanel exerts its broad-spectrum antiepileptic effects is not fully understood.

Previous studies have demonstrated that perampanel produces a concentration- and time-dependent inhibition of *I*_Na_ in hippocampal mHippoE-14 neurons. Notably, perampanel preferentially suppresses the late component of *I*_Na_ compared to the peak component, with reported IC_50_ values of 0.78 μM and 4.12 μM, respectively. This corresponds to a 5.3-fold selectivity for late versus peak *I*_Na_ inhibition [10]. These findings also suggest that perampanel effectively targets the non-inactivating *I*_Na_ [23,24]. Therefore, in addition to its established antagonism of AMPA receptors, perampanel appears to suppress *I*_Na_ through a distinct, noncanonical mechanism, which may further contribute to the reduction in neuronal and glioma cell excitability in vivo [10,11,24,25,27,30,31,32,33,34,35].

**Figure 1:**
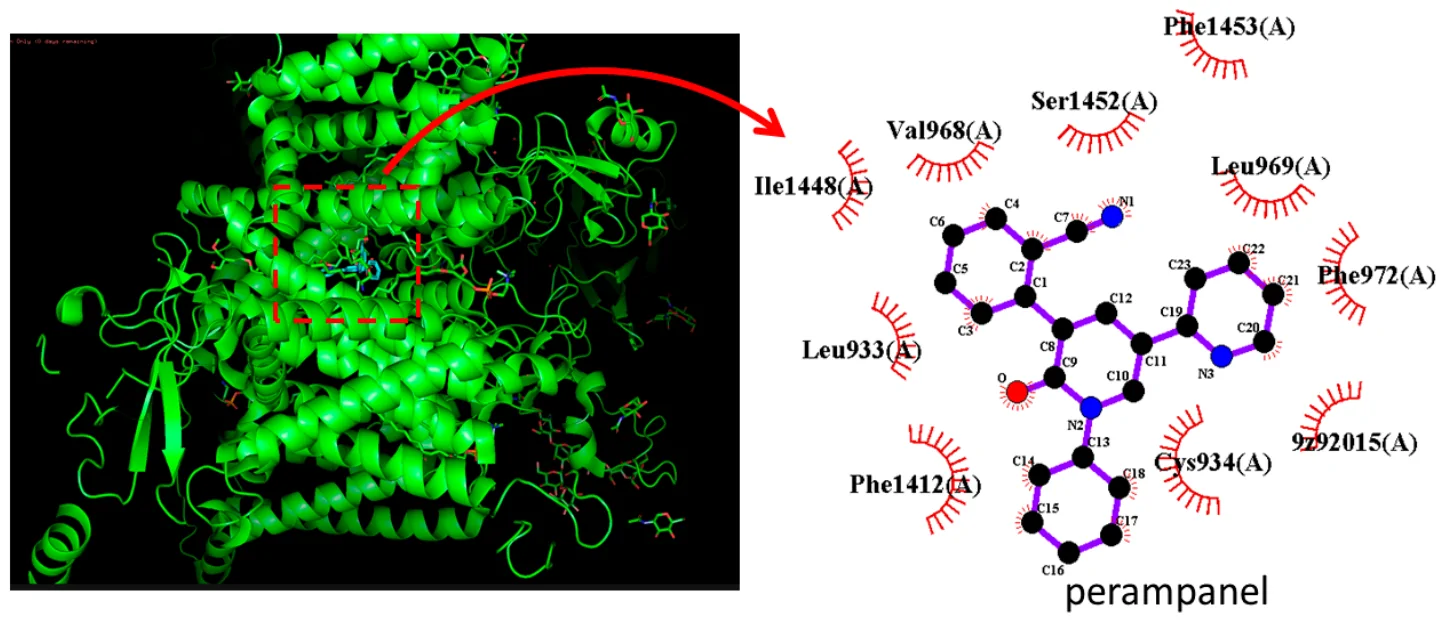
Docking interaction between the perampanel molecule and the human Na_V_1.6 (or SCN8A) channel. The (**left**) graph represents the docking interaction between perampanel and the Na_V_1.6 channel using PyRx 0.8 software. The protein structure of Na_V_1.6 was obtained from the Protein Data Bank (PDB ID: 9DK5), and the three-dimensional chemical structure of perampanel was retrieved from PubChem (Compound CID: 9924495). On the (**left**), the red dashed box highlights a snapshot of perampanel engaging in hydrophobic interactions with the channel, which is enlarged and illustrated on the (**right**). Of note, in this and the following figures showing predicted docking, the red arcs, with spokes pointing toward the molecule, denote hydrophobic contacts, and the corresponding graph on the right, which was generated by LigPlot^+^ v2.3, indicates an expanded view of the red dashed box with a curved arrow in the left panel. “9z92015” shown in the right panel does not represent an amino acid itself. Instead, it is a way of identifying a residue within a specific chain of the protein structure. In this figure and all subsequent docking figures, capital letters shown in parentheses indicate the corresponding protein chain identifiers. The XYZ axes are located at the coordinates (173, 164, 167).

We further conducted a detailed analysis of the atomic interaction between the Na_V_1.6 protein and perampanel using PyRx 0.8 software, employing its embedded AutoDock (PyRx 0.8; AutoDock Vina 11.2) function. The procedure for molecular docking is shown in the Appendix A. Figure 1 illustrates the predicted docking sites of the perampanel molecule. Notably, during the docking analysis with the Na_V_1.6 channel, perampanel was observed to exhibit hydrophobic interactions with several residues, including Cys934(A), Val968(A), Leu969(A), Phe972(A), Phe1412(A), Ile1448(A), Ser1452(A), and Phe1453(A). These findings suggest that perampanel exhibits binding affinity for amino acid residues within the Na_V_1.6 channel, with an estimated binding energy of −8.50 kcal/mol. The interaction predominantly occurs within the transmembrane segment of chain A in the Na_V_1.6 channel. Therefore, the perampanel-induced changes in *I*_Na_ amplitude and gating kinetics observed in glioma cells may be associated, at least in part, with its effect on Na_V_1.6 channels, rather than being solely attributable to AMPA receptor inhibition [10,33].

**Figure 2:**
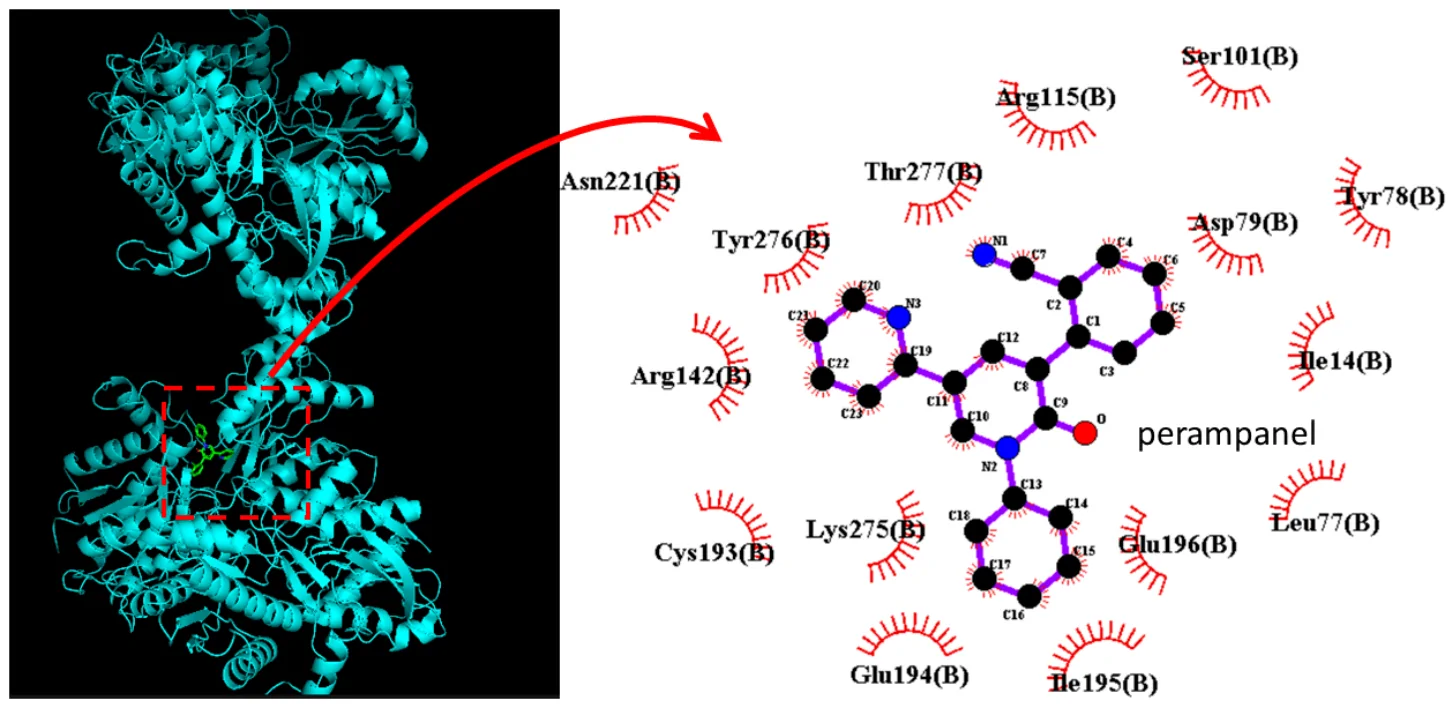
Molecular docking analysis of perampanel with the AMPA receptor. The protein structure of the AMPA receptor was acquired from the PDB (PDB: 9XJM). The structure of the AMPA receptor was docked with the perampanel molecule using PyRX software, as indicated in the (**left**) panel. The diagram of the interaction between the AMPA receptor protein and the perampanel molecule was generated by LigPlot^+^ (**right** side). The (**right**) panel shows an enlarged view of the region outlined by the red dashed box in the (**left**) panel, as indicated by the curved arrow. The XYZ axes are located at the coordinates (218, 218, 219).

Previous reports have demonstrated the ability of perampanel to bind to the AMPA receptor [28]. We also conducted a predicted docking analysis between perampanel and the AMPA receptor. A docking analysis of the AMPA receptor with the perampanel molecule was performed. The protein structure of the AMPA receptor was obtained from the PDB (PDB ID: 9XJM). The predicted docking sites of the perampanel molecule, with which the amino acid residues can interact, are presented in Figure 2. Notably, the perampanel molecule was predicted to form hydrophobic contacts with certain residues, including Ile14(B), Leu77(B), Tyr78(B), Asp79(B), Ser101(B), Arg115(B), Arg142(B), Cys193(B), Glu194(B), Ile195(B), Glu196(B), Asn221(B), Lys275(B), Tyr276(B), and Thr277(B). These docking results suggest that perampanel can bind to the AMPA receptor with a binding affinity of −7.7 kcal/mol. The docking results are preliminary and hypothesis-generating, warranting further experimental validation before any definitive translational conclusions can be drawn. The therapeutic use of perampanel in brain tumors is likely to represent a paradigm shift in managing glioma-associated seizures, as it simultaneously targets both AMPA-type receptors and Na_V_ channels.

### 2.2. Valproic Acid

Valproic acid (2-*n*-propylpentanoic acid, Depakine^®^, Convulex^®^), one of the most common histone deacetylase inhibitors [35], is a medication primarily used to treat epilepsy and bipolar disorder and to prevent migraine headaches. This drug has been demonstrated to be beneficial for neuropathic pain and fibromyalgia in adults [36,37,38,39]. It has been reported to block *I*_Na_ [40,41,42]. Furthermore, valproic acid has been recently shown to be a promising therapeutic agent in the treatment of glioma [43,44].

Valproic acid-mediated inhibition of *I*_Na_ can be counteracted by the subsequent addition of tefluthrin. Consistent with previous findings, tefluthrin—a pyrethroid insecticide—has been reported to enhance *I*_Na_ across multiple cell types [24,45,46]. These findings therefore suggest that U87 glioma cells express physiologically active *I*_Na_, which can be inhibited by valproic acid and stimulated by tefluthrin. The non-inactivating (persistent) *I*_Na_ elicited by an ascending ramp pulse is also inhibited by valproic acid. The abnormal and aberrant electrical activity associated with glioma-related epilepsy is closely linked to Na_V_ channel activity [10,12,24,25,26,47] and can therefore be controlled by valproic acid through the inhibition of *I*_Na_.

The influence of valproic acid likely extends beyond the inhibition of histone deacetylase inside the cells. In addition to its inhibitory effect on histone deacetylase in glioma cells, the suppressive action of valproic acid on Na_V_ channels in these cells may also play an important role [12]. It is worth noting that the primary function of histone deacetylase is to deacetylate histone proteins [31]; therefore, its action mainly occurs within the cell nucleus, whereas Na_V_ channels function at the cell surface membrane [21,22,26,48]. The concentration of valproic acid required to reach and exert effects within the cell nucleus would likely be substantially higher than that needed to act on the cell surface membrane. Furthermore, because valproic acid acts directly on ion channels in the plasma membrane, its effects can develop much more rapidly than its nuclear actions, which typically involve transcriptional and epigenetic regulation. Consequently, ion channel modulation is expected to occur on a substantially faster timescale than downstream intracellular responses [25,26,38,39,48]. Bridging this temporal gap remains an important objective, as it may help establish ion channel activity as an early initiating event that triggers subsequent intracellular signaling processes, including nuclear responses. Furthermore, the extent to which valproic acid-induced inhibition of *I*_Na_ influences glioma cell invasiveness and migration warrants further investigation, particularly in the context of drug-repurposing strategies [12,26,41,42].

## 3. Large-Conductance Ca^2+^-Activated K^+^ (BK_Ca_) Channel

Large-conductance Ca^2+^-activated K^+^ (BK_Ca_) channels, also referred to as BK, Maxi-K, K_Ca_1.1, KCNMA1, or Slo1, belong to the family of voltage-gated K^+^ channels. These channels are activated by elevations in intracellular Ca^2+^ levels, membrane depolarization, or the synergistic action of both stimuli [13,49,50,51,52]. Upon activation, BK_Ca_ channels facilitate a robust efflux of K^+^ ions across the plasma membrane and exhibit pronounced outward rectification. A hallmark feature of BK_Ca_ channels is their exceptionally high single-channel conductance, typically ranging from 150 to 250 pS, which distinguishes them from other K^+^ channels and accounts for their designation as “Maxi-K” or large-conductance K^+^ channels [49,50,51,52]. Several lines of evidence indicate that this family of K^+^ channels is functionally expressed in glioma cells [14,51,52,53,54,55]. Moreover, altered BK_Ca_ channel activity has been shown to modulate glioma cell behavior, suggesting a potential role in glioma progression and pathophysiology [51,53,54,55,56].

Previous reports have demonstrated that BK_Ca_ channel activity in cardiac fibroblasts can influence adjacent cardiac cells through effective electrical coupling, thereby modulating the overall electrical excitability of the heart [50,57]. Glioma cells are able to infiltrate surrounding brain tissue in complex ways. Similarly, BK_Ca_ channels expressed in glial and glioma cells may modulate the electrical activity of adjacent neurons by influencing intercellular electrical signaling, either through ephaptic interactions or via neuro-glioma synaptic communication pathways [58,59,60].

### Cilostazol

Cilostazol (6-[4-(1-cyclohexyl-1H-tetrazol-5-yl)butoxy]-3,4-dihydro-2(1H)-quinolinone, Pletal^®^, Pletaal^®^) is a thrombolytic and vasodilator drug that was originally developed as a selective inhibitor of cyclic nucleotide phosphodiesterase type 3A (PDE3A) [61]. In addition to its PDE3A inhibitory activity, cilostazol has also been reported to inhibit adenosine uptake in various cell types [62]. Previous studies have demonstrated its ability to stimulate Ca^2+^-activated K^+^ currents (*I*_K(Ca]_) in human neuroblastoma SK-N-SH cells [63]. Moreover, this drug has been reported to enhance the activity of BK_Ca_ channels in pituitary tumor (GH_3_) cells and pheochromocytoma (PC12) cells [13]. The cilostazol-mediated increase in channel open probability is linked to the prolongation of the mean open time of the channel [13].

An earlier study demonstrated that cilostazol enhances BK_Ca_ channel activity in radiation-resistant glioma cells [14]. Restoration of BK_Ca_ channel function by cilostazol was shown to suppress radiation-resistance-induced cell migration, proliferation, tumor-sphere formation, and in vivo growth. Accordingly, cilostazol has considerable potential as an adjuvant therapy in combination with conventional radiotherapy, chemotherapy, or radiochemotherapy for glioblastoma multiforme, the most malignant form of glioma [14].

The BK_Ca_ channels in glioma cells can be sensitive to an increase in the presence of cilostazol; subsequent addition of paxilline can attenuate the cilostazol-induced decrease in channel activity. Paxilline is known to suppress the activity of BK_Ca_ channels effectively in glioma cells [54,64,65]. These results indicate that BK_Ca_ channels are functionally expressed in these cells, as demonstrated by their responsiveness to cilostazol and sensitivity to blockade by paxilline [13,14,54,55,63]. However, given that cell-attached recordings preserve intracellular signaling, parallel, cytosolic-mediated pathways—such as phosphodiesterase inhibition—cannot be definitively excluded.

**Figure 3:**
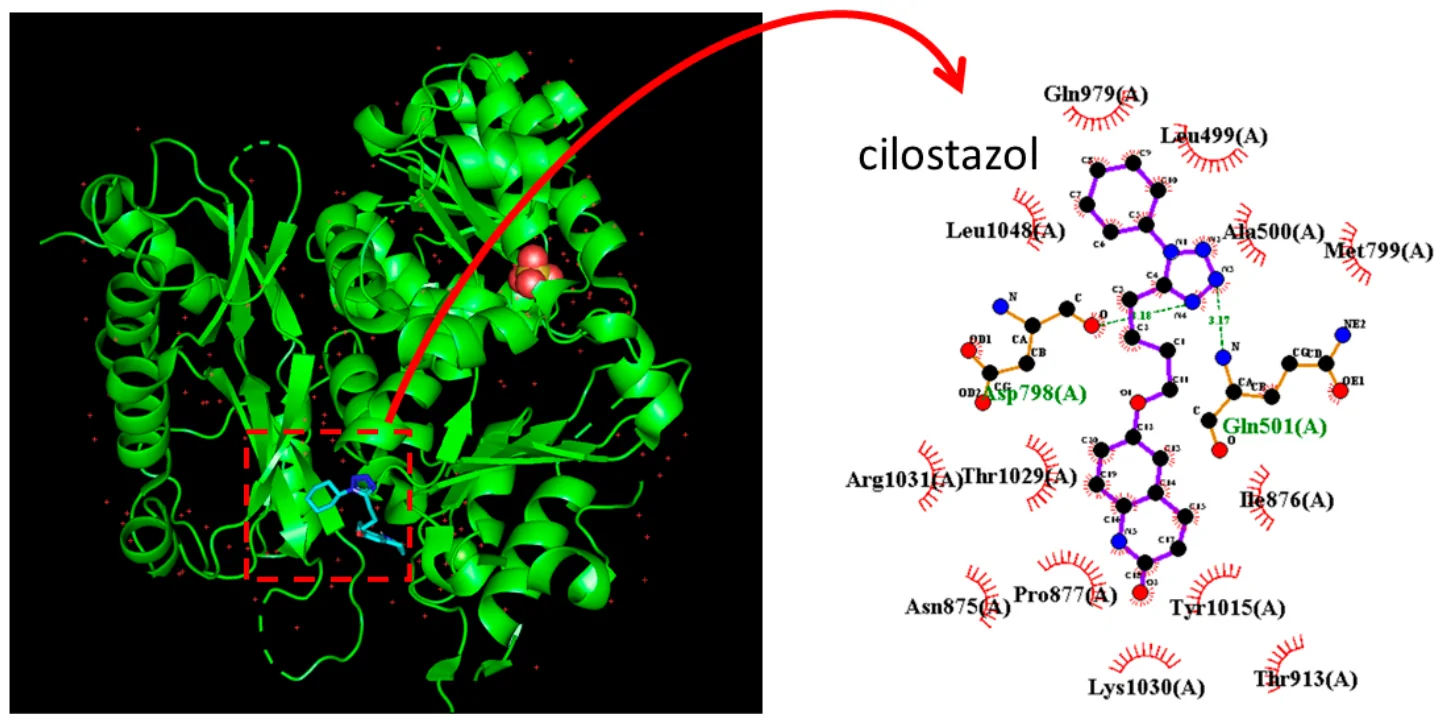
Molecular docking analysis of cilostazol with the human BK_Ca_ (KCNMA1/K_Ca_1.1) channel. The protein structure was retrieved from the PDB (PDB ID: 6V5A), and the three-dimensional structure of cilostazol was obtained from PubChem (Compound CID: 2754). The BK_Ca_ channel structure was subjected to molecular docking with cilostazol using PyRx software, as illustrated in the (**left**) panel. The diagram of the interaction between the BK_Ca_ channel protein and cilostazol was generated by LigPlot^+^ (**right** side). The small red dots represent water molecules within the structure, with a total of 226 atoms in this group. The XYZ axes are located at the coordinates (43, 42, 18).

Given the pronounced stimulatory effect of cilostazol on BK_Ca_ channels in glioma cells [14], we further employed molecular docking analysis to explore its potential binding interactions with the BK_Ca_ channel protein at the atomic level. The docking procedure was performed using PyRx software to identify the most energetically favorable binding pose of cilostazol within the channel protein. The protein structure of the BK_Ca_ channel, determined by X-ray crystallography at a resolution of 2.00 Å, was retrieved from the PDB (PDB ID: 6V5A). The predicted docking sites of the cilostazol molecule and its potential interaction with specific amino acid residues are illustrated in Figure 3. Notably, the cilostazol molecule was predicted to establish hydrophobic contacts with certain residues, including Leu499(A), Ala500(A), Met799(A), Asn875(A), Ile876(A), Pro877(A), Thr913(A), Gln979(A), Tyr1015(A), Thr1029(A), Lys1030(A), Arg1031(A), and Leu1048(A). Cilostazol was also predicted to form hydrogen bonds with Asp798(A) and Gln501(A), with bond distances of 3.18 Å and 3.17 Å, respectively. Molecular docking analysis predicted that cilostazol interacts with the BK_Ca_ channel protein with an estimated binding affinity of −8.0 kcal/mol. In the high-resolution X-ray crystal structure (2.00 Å), several water molecules remained associated with the channel protein and may contribute to the stability of the protein–ligand complex. Consistent with previous studies [13,14], these findings suggest that the BK_Ca_ channel is susceptible to modification by cilostazol. Consequently, cilostazol, an approved therapeutic agent for cardiovascular disorders, may represent a promising candidate for drug repurposing in glioma treatment.

## 4. Intermediate-Conductance Ca^2+^-Activated K^+^ (IK_Ca_) Channel

The IK_Ca_ channels (also known as K_Ca_3.1, SK4, IKCa1, and KCNN4) are encoded by the KCNN4 gene. They have been cloned from human, mouse, and rat tissues, and their mechanisms linked to hormonal secretion, cell motility, cell proliferation, and Ca^2+^ influx and K^+^ efflux regulation have been extensively studied in many non-excitable or neoplastic cells [6,16,66,67]. These channels have a single-channel conductance of 20–60 pS, and the pharmacological profiles of IK_Ca_ channels are quite distinct from those of large- and small-conductance Ca^2+^-activated K^+^ channels [6,67,68]. The modulators of IK_Ca_ channels represent a potential therapeutic approach to a variety of diseases, including malignant gliomas [5,56,69].

### 4.1. Arecoline

Areca nut has long been used as a medical and psychoactive stimulant in China and is the leading cause of oral cancer there [70]. Arecoline (1,2,5,6-tetrahydrol-1-methyl-3-pyridinecarboxylic acid methyl ester, methyl 1-methyl-1,2,5,6-tetrahydropyridine-3-carboxylate) is an alkaloid extracted from the areca nut, functions as a non-selective agonist of muscarinic receptors, and has been shown to have carcinogenicity, cytotoxicity, and immunotoxicity [71].

Previous reports have demonstrated that arecoline is effective in suppressing the activity of IK_Ca_ channels in glioma (U373 and U87MG) cells [15]. In U373 cells, arecoline was found to induce a time-, concentration-, and state-dependent prolongation of the mean closed time of IK_Ca_ channels with no clear change in mean open time. Arecoline was also found to decrease the activity of inwardly rectifying K^+^ (K_IR_) channels in these glioma cells [19]. Arecoline-induced suppression of these K^+^ channels, accompanied by membrane depolarization, is a rapid and direct action that is independent of muscarinic receptor binding [15,19].

Previous studies have demonstrated the expression of K_Ca_3.1 mRNA in both U373 and U87MG glioma cells, whereas exposure to arecoline did not alter K_Ca_3.1 transcript levels [15]. These findings suggest that the inhibitory action of arecoline on K_Ca_3.1 channels is unlikely to result from changes in gene expression but rather from a direct modulation of channel activity at the plasma membrane. Given the recognized involvement of K_Ca_3.1 channels in glioma progression and therapeutic resistance [67], pharmacological blockade of these channels by arecoline may provide a valuable strategy for potentiating the anti-tumor efficacy of conventional cytotoxic agents. Consequently, arecoline or related K_Ca_3.1-targeting compounds may hold promise as adjunctive therapeutic agents for the treatment of malignant gliomas and other neoplastic disorders [6,64,66,67].

### 4.2. Berberine

Berberine (9,10-dimethoxy-5,6-dihydro [1,3]dioxolo [4,5-g]isoquino [3,2-a]isoquinolin-7-ium) is an isoquinoline alkaloid found in numerous medicinal plants, particularly within the genera *Berberis* (Berberidaceae) and *Coptis* (Ranunculaceae). Although berberine is not typically marketed as a prescription medication in most countries, it is widely sold as a dietary supplement ingredient. Extracts from these plants have been widely used in traditional medicine to treat conditions such as jaundice, dysentery, hypertension, and various other ailments [16,72]. Moreover, berberine itself has demonstrated notable anti-neoplastic activities against glioma [73].

Previous studies have demonstrated that berberine directly exerts inhibitory effects on the activity of IK_Ca_ channels in human myeloma cells [16]. Berberine also decreases the open probability of IK_Ca_ channels without altering the single-channel amplitude. Consistent with these electrophysiological observations, berberine markedly suppresses the proliferation of neoplastic plasma cells. These findings suggest that berberine is a biologically active compound with considerable therapeutic potential for the treatment of various diseases, including glioma [72,73,74,75,76,77]. Beyond its ability to modulate intracellular signaling pathways [74,75,76,77], berberine and other structural analogs may exert pharmacological efficacy through direct interaction with membrane K^+^ channels, which could represent a key underlying mechanism of their biological actions [16,78,79].

**Figure 4:**
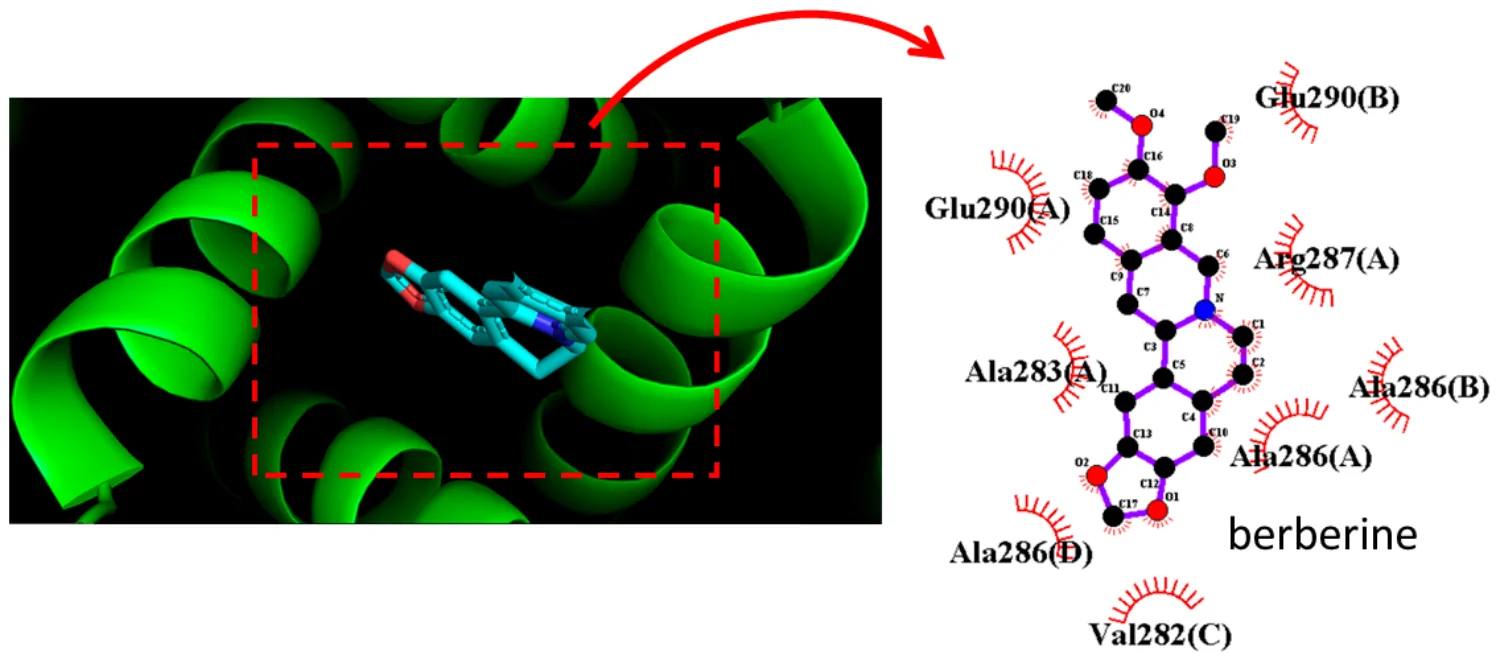
Predicted docking between the human IK_Ca_ (KCNN4, K_Ca_3.1) channel and the berberine molecule. The (**left**) panel depicts the channel protein acquired from the PDB (PDB ID: 9ZPT) and the three-dimensional structure of berberine from PubChem (Compound CID: 2353). PyRx software was used to demonstrate that the IK_Ca_ channel structure can be optimally docked with the berberine molecule. The (**right**) panel depicts an expansion of the red dashed box with a curved arrow in the (**left**) panel. The XYZ axes are located at the coordinates (215, 215, 220). The schematic illustrating the interaction between the IK_Ca_ channel and the berberine molecule was generated using LigPlot^+^.

Berberine is a tetracyclic isoquinoline alkaloid with a planar, positively charged quaternary ammonium structure. It exhibits limited aqueous solubility and relatively constrained membrane permeability due to its cationic nature. To examine how berberine interacts with the IK_Ca_ channel protein, we used the protein structure from the PDB (PDB ID: 9ZPT). PyRx 0.8 software was used to determine how the berberine molecule docks into the channel protein. Figure 4 presents the predicted docking sites of berberine for interaction with amino acid residues of the channel. Specifically, berberine was predicted to establish hydrophobic interactions with residues Val282(C), Ala283(A), Ala286(A/B/D), Arg287(A), and Glu290(A/B), with a binding affinity of −5.6 kcal/mol. Accordingly, berberine is anticipated to interact with the IK_Ca_ channels and thereby modulate voltage-gated K^+^ currents (*I*_K_) in glioma cells.

### 4.3. Oxaliplatin

Glioblastoma multiforme is a cancer with a dismal prognosis, and the relatively short life expectancy of affected patients remains disappointing despite recent extensive advances in treatment [1,80,81]. Ionic currents inherent in malignant glioma cells have been increasingly reported to influence the behavior of these cells [2,3,31]. Specifically, numerous reports have demonstrated that any perturbation in the functional expression of IK_Ca_ channels enriched in glioma cells or other types of neoplastic cells can interfere with invasiveness or progression of malignant tumors [65,66,81,82].

Oxaliplatin (oxalato(trans-l-1,2-diaminocyclohexane)platinum(II), Eloxatin^®^, Orectalip^®^, Oxalip^®^) belongs to a family of platinum-based chemotherapeutic compounds and is one of the most active drugs against many types of solid tumors, including gliomas [83,84]. Previous reports have shown that oxaliplatin or other structurally similar compounds can interact with IK_Ca_ channels to decrease the amplitude of *I*_K_ in glioma cells [17].

In human 13-06-MG glioma cells, oxaliplatin was shown to decrease the amplitude of *I*_K_, and 5,6-dichloro-1-ethyl-1,3-dihydro-2H-benzimidazol-2-one (DCEBIO) or ionomycin can reverse oxaliplatin-induced inhibition of *I*_K_. DCEBIO has been reported to stimulate the activity of IK_Ca_ channels [17,85]. Oxaliplatin also diminishes the activity of IK_Ca_ channels in a concentration-dependent manner with an IC_50_ of approximately 67 μM, suggesting that it can stabilize the channel in a closed state without changing the single-channel conductance of these channels [17]. Therefore, the oxaliplatin-mediated effect on IK_Ca_ channel activity may potentially influence the malignant behavior of glioma cells, provided similar in vivo actions exist.

### 4.4. Temozolomide

Temozolomide (3-methyl-4-oxoimidazo [5,1-d][1,2,3,5]tetrazine-8-carboxamide, Temodar^®^, Temodal^®^) is an oral alkylator of the imidazotetrazine family. It was originally considered a small-molecule prodrug that is readily absorbed through the gastrointestinal tract and is regarded as one of the most effective pharmacological treatments for gliomas [86]. This drug was thought to be cytotoxic because 5-(3-methyltriazen-l-yl)imidazole-4-carboxamide alkylates DNA at the O^6^ and N^7^ positions of guanine [86]. O^6^-methylguanine DNA methyltransferase (MGMT) was thought to be an important factor that influences the cellular response to temozolomide [86]. However, it has been demonstrated that patients without MGMT methylation can also benefit from temozolomide treatment [87,88]. Previous studies suggested that temozolomide treatment may contribute to a reduction in seizure frequency in patients with low-grade gliomas [87,88].

**Figure 5:**
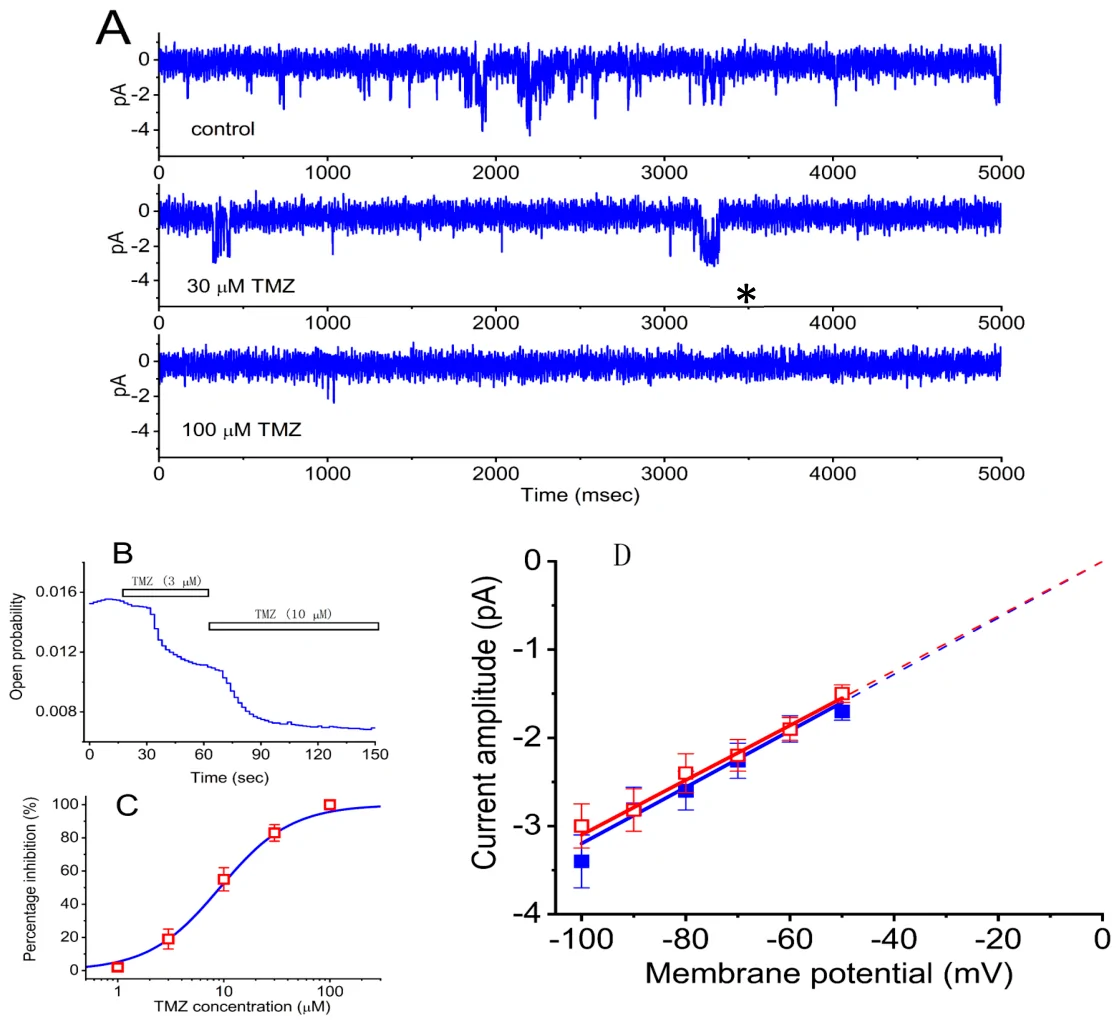
Concentration-dependent inhibition of IK_Ca_ channel activity by temozolomide (TMZ) in U373 glioma cells. Cells were immersed in a high-K^+^ solution (145 mM K^+^) containing 1.8 mM CaCl_2_, while the holding potential was maintained at −70 mV. The recording pipettes were filled with a K^+^-rich solution. IK_Ca_ channel activity was assessed using the cell-attached patch-clamp configuration in the absence and presence of different concentrations of temozolomide. (**A**) Representative blue current traces illustrating IK_Ca_ channel activity under control conditions and after exposure to 30 or 100 μM temozolomide. Downward current deflections in current (as indicated by an asterisk) represent channel-opening events. The *x*-axis labeling of the lower panel was applied to the upper and middle panels. (**B**) Time course of temozolomide-induced inhibition of IK_Ca_ channel open probability at −70 mV. Temozolomide (TMZ), at concentrations of 3 and 10 μM, was applied throughout the periods indicated by the horizontal open bars. (**C**) Concentration–response relationship for temozolomide-mediated inhibition of IK_Ca_ channels. Cells were exposed to temozolomide (TMZ) at concentrations ranging from 1 to 100 μM. Data are presented as mean ± SEM, with sample sizes ranging from 9 to 13 observations for each concentration. The smooth blue sigmoidal curve represents the best-fit regression obtained using the modified Hill equation. The estimated IC_50_ value was 9.2 μM, with a maximal inhibition of 100% and a Hill coefficient of 1.2. (**D**) Current–voltage (I-V) relationships of IK_Ca_ channels recorded under control conditions (blue filled squares) and following exposure to 10 μM temozolomide (red open squares). Data are expressed as mean ± SEM, with sample sizes ranging from 9 to 12 observations for each data point. The dashed lines represent extrapolated trends, whose intersection with the voltage axis indicates the reversal potential of approximately 0 mV. Exposure to temozolomide did not significantly alter the single-channel conductance of IK_Ca_ channels in U373 glioma cells. This figure is adapted from Yeh et al. [18] and is distributed under the Creative Commons Attribution (CC BY) license.

As shown in Figure 5, the addition of temozolomide to the bath medium significantly decreased IK_Ca_ channel activity by reducing channel opening [18]. Under control conditions, the open probability of IK_Ca_ channels at −70 mV was 0.014 ± 0.003 (*n* = 9). One minute after temozolomide (10 μM) had been added to the bath, the open probability was significantly (*p* < 0.05) lower: 0.0084 ± 0.0009 (*n* = 9). After the cells had been washed to eliminate the added temozolomide, the channel activity rose to 0.011 ± 0.003 (*n* = 6). The amplitudes of the single-channel currents were unaltered in the presence of temozolomide. After temozolomide treatment (1–100 μM), the probability of open IK_Ca_ channels was noted to be concentration-dependent (Figure 5A–C).

The half-maximal concentration (IC_50_) needed for temozolomide to inhibit IK_Ca_ channel activity was 9.2 μM. Temozolomide treatment affecting IK_Ca_ channels at different membrane potentials was also evaluated. The plots of current amplitude as a function of holding potential were constructed. The single-channel conductance of IK_Ca_ channels calculated from the linear current–voltage (I-V) relationship in control was 32 ± 4 pS (*n* = 9) with a reversal potential of 0 ± 2 mV (*n* = 9) (Figure 5D). However, single-channel slope conductance (31 ± 4 pS; *n* = 9, *p* > 0.05) of IK_Ca_ channels was unchanged after temozolomide (10 μM) treatment. These results indicate that temozolomide effectively suppresses the activity of IK_Ca_ channels in U373 glioma cells [18]. Therefore, the inhibitory effect of temozolomide on IK_Ca_ channel activity is likely associated with an interaction with the Ca^2+^-activated K^+^ (K_Ca_) channel protein in glioma cells [18].

**Figure 6:**
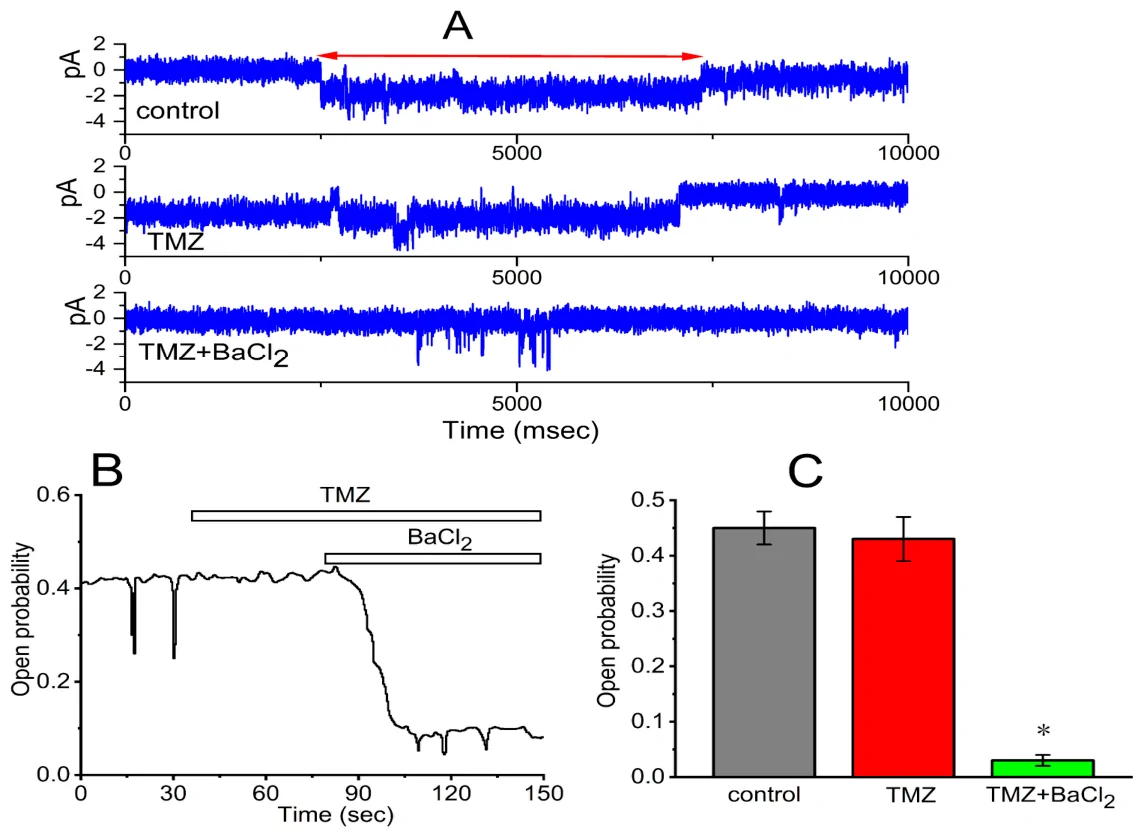
Temozolomide does not alter Kir channel activity in U373 cells under Ca^2+^-free conditions. Cell-attached patch-clamp recordings were obtained from U373 cells bathed in Ca^2+^-free Tyrode’s solution, with the membrane potential held at 0 mV relative to the bath. (**A**) Representative blue current traces recorded under control conditions (upper), after application of 30 μM temozolomide (TMZ, middle), and following co-application of 30 μM temozolomide and 1 mM BaCl_2_ (lower). Downward current deflections indicate channel open events. The double horizontal red arrow indicates an open event of the Kir channel, with an open duration of approximately 4 s. The *x*-axis labeling of the lower panel was applied to the upper and middle panels. (**B**) Time-dependent changes in Kir channel open probability at 0 mV (relative to the bath) following temozolomide (TMZ) application. Horizontal open bars denote the intervals during which temozolomide or BaCl_2_ was present in the bath solution. (**C**) Summary of the experimental data expressed as mean ± SEM, with sample sizes ranging from 9 to 10 observations for each bar. * Significantly different from the temozolomide (TMZ) alone group (*p* < 0.05). This figure is adapted from Yeh et al. [18] and is distributed under the Creative Commons Attribution (CC BY) license.

The effects of temozolomide on the inwardly rectifying K^+^ (Kir) channels in U373 cells were further investigated. Cells were immersed in Ca^2+^-free Tyrode’s solution, and when the membrane potential was held at 0 mV relative to the bath, distinct Kir channel activity could be readily observed (Figure 6). It should be noted that Kir channels are distinguished by their inward rectification, displaying unusually long open times that can persist for several seconds. In practical terms, this means that their semiconductor-like properties facilitate the influx of K^+^ ions into the cell while restricting their efflux. The addition of temozolomide (30 μM) alone to the bath, however, did not produce any significant change in Kir channel activity in these cells. In the continued presence of temozolomide, subsequent application of BaCl_2_ (1 mM) almost completely suppressed the channel activity by markedly reducing the channel open probability. These results indicate that temozolomide does not inhibit Kir channels in U373 cells, although these channels remain sensitive to suppression by BaCl_2_ [18]. However, the study did not report the MGMT methylation status of the analyzed cell lines. Additional experimental and clinical studies are needed to determine whether these observations can be generalized to MGMT-positive gliomas.

## 5. Inwardly Rectifying K^+^ (Kir) Channel

The inwardly rectifying K^+^ (Kir) channel is a specialized membrane ion channel that preferentially allows K^+^ ions to move into a cell rather than out of it under many electrical conditions. These channels are fundamental for maintaining the resting membrane potential, controlling cellular excitability, stabilizing cardiac rhythm, regulating insulin secretion, and shaping neuronal signaling [89,90]. The Kir2.1 channel, which belongs to the inwardly rectifying K^+^ (Kir) channel family, displays characteristic inward rectification and is critically involved in stabilizing the resting membrane potential in various cell types, including neurons, microglia and glioma cells [89,90,91,92].

In addition, Kir channel activity is highly sensitive to inhibition by extracellular barium (Ba^2+^) ions [18,19,93] (Figure 6). In particular, altered Kir2.1 channel activity has been shown to affect the proliferation and invasiveness of glioma cells [91]. Moreover, loss-of-function of the Kir2.1 channel has been reported to impair glioma cell proliferation and invasion [92,94]. In addition to the expression of the Kir2.1 channel in glioma, Kir2.2 and Kir2.3 channels were also reported to be expressed, although to a lesser extent.

### Triptolide

Triptolide, a diterpene triepoxide isolated from *Tripterygium wilfordii* Hook F.—commonly known as the “Thunder God Vine” or “Chinese Thunder God Vine”—is a bioactive compound with remarkable pharmacological properties. It demonstrates strong anticancer, anti-inflammatory, and immunosuppressive effects [95,96,97,98]. Notably, triptolide has been shown to protect central nervous system neurons and stimulate axon growth in dopaminergic neurons [96].

In addition, it suppresses the proliferation and invasion of malignant glioma cells [97,98] while inducing apoptosis [99], highlighting its potential as a therapeutic agent in neurodegenerative diseases and cancer treatment. Because of its small size and high lipid solubility, triptolide can cross the blood–brain barrier and produce significant effects on glial or glioma cells [98,99,100,101,102].

**Figure 7:**
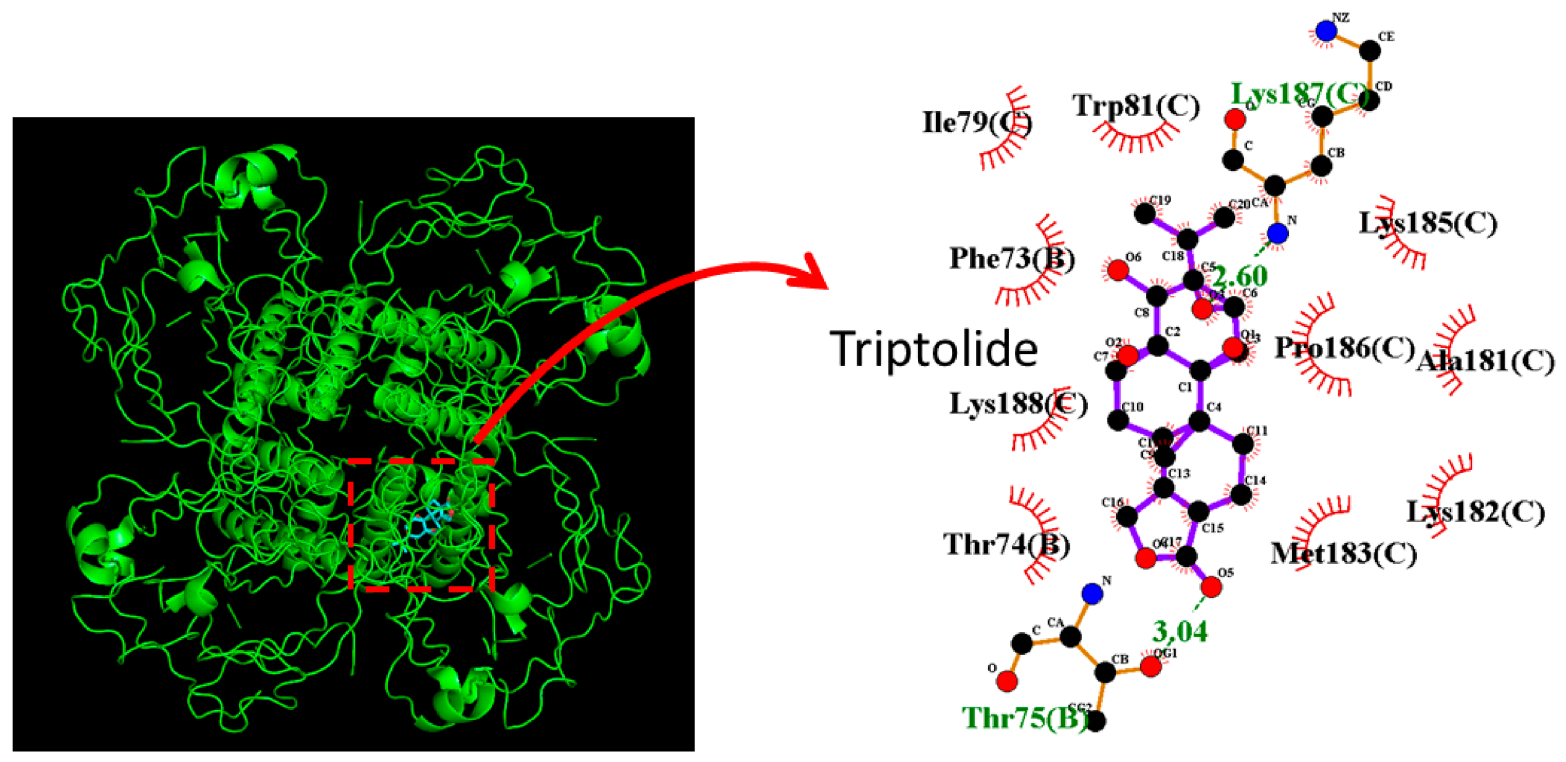
Docking interaction between the triptolide molecule and the KCNJ2 (or Kir2.1-encoded) channel. The left graph refers to the docking prediction between triptolide and the KCNJ2 channel. The channel protein structure was obtained from the PDB (PDB ID: 8QQL), and the chemical structure of triptolide was obtained from PubChem (Compound CID: 107985). On the (**left**), the red dashed box highlights a snapshot of triptolide engaging hydrophobic interactions and forming hydrogen bonds with the channel, which is enlarged and illustrated on the (**right**). The green dashed line indicates hydrogen bond formation, with bond lengths estimated at 2.60 and 3.04 Å. The XYZ axes are located at the coordinates (102, 102, 107). The corresponding graph in the (**right**) panel indicates an expanded view of the red dashed box with a curved arrow in the (**left**) panel.

To examine how triptolide interacts with the Kir channel protein, we used the structure of the KCNJ2 protein from the PDB. Previous reports showed the presence of KCNJ2 channels in glioma cells [92]. The KCNJ2 channel protein structure was acquired from the PDB (PDB ID: 8QQL), and a structure-based virtual screen was performed. Figure 7 presents the predicted docking sites of triptolide for interaction with amino acid residues of the KCNJ2 channel. The triptolide molecule was predicted to form hydrophobic contacts with residues Phe73(B), Thr74(B), Ile79(C), Trp81(C), Ala181(C), Met183(C), Lys182(C), Lys185(C), Pro186(C), and Lys188(C), as well as hydrogen bonds with residues Thr75(B) and Lys187(C), with estimated distances of 3.04 and 2.60 Å, respectively. It is therefore plausible that triptolide interacts with KCNJ2 channels, thereby modulating the open probability and gating kinetics of Kir channels in glioma cells; however, this hypothesis remains to be experimentally validated, for example, in cell-based assays. Therefore, triptolide-induced inhibition of Kir channels is likely to be closely linked to a wide range of its biological activities.

## 6. Conclusions and Future Perspectives

Growing evidence has demonstrated that neoplastic cells across various glioma subtypes express physiologically active Na_V_ channels, contributing to aberrant excitability [10,24,25,26,27,58,102,103]. Alongside dysregulated excitatory transmitters such as glutamate, this abnormal channel activity represents a key mechanism underlying tumor-associated epilepsy; aberrant excitability of Na_V_ channels on the tumor cell membrane also represents an important factor in tumor-related epilepsy [24,25]. Consequently, antiepileptic agents, including perampanel and valproic acid, are expected to provide therapeutic benefits in managing these convulsive disorders [26,46,47,48,87,104,105,106,107,108]. Interestingly, several studies also suggest that Na_V_ channel activators may exert anti-tumor effects in gliomas [7,9], highlighting a complex dual role. The extent to which small-molecule modulators alter voltage-dependent hysteresis of *I*_Na_ in relation to glioma malignancy remains an open question, warranting further investigation [49,108]. Voltage-dependent hysteresis of *I*_Na_ in response to an isosceles-triangular ramp pulse refers to the non-linear, history-dependent behavior of Na_V_ channels, where the current–voltage (I-V) relationship forms a loop (rather than a single curve) when the membrane potential is swept up and down in a triangular fashion. This hysteresis reflects the dynamic gating properties of Na_V_ channels and is often visualized as a figure-of-eight pattern with distinct low- and high-threshold loops [49,108].

In the glioma tumor microenvironment, extracellular K^+^ concentrations serve as important regulatory factors. Increased activity of plasmalemmal K^+^ channels may facilitate substantial efflux of K^+^ from tumor cells, thereby modifying extracellular K^+^ levels. Because the extracellular space within gliomas is markedly restricted and intracranial volume is confined by the skull, this efflux leads to elevated extracellular K^+^ concentrations and contributes to tumor cell shrinkage [109,110]. Such aberrant changes in the K^+^ equilibrium potential alter the membrane excitability of neighboring cells [19,88,107,109]. Therefore, selective blockade of K^+^ channels, by reducing excessive intracellular K^+^ efflux, may help stabilize glioma-associated excitability, potentially through modulation of ephaptic coupling mechanisms [58,59,109,110].

On the other hand, when K^+^ channels are highly active near the resting membrane potential, further increases in their activity induce membrane hyperpolarization. Membrane hyperpolarization itself has also recently been noted to alter the availability of Na_V_ channels [23], thereby reducing membrane excitability. Increasing evidence suggests that glioma-derived exosomes participate in intercellular communication by transferring bioactive cargoes that modulate the expression and activity of ion channels, thereby influencing glioma progression and the surrounding microenvironment [5,78,109,110,111].

Previous studies have suggested the potential presence of voltage-gated Ca^2+^ (Ca_V_) channels in glioma cells [111,112]. However, most of these investigations relied primarily on Western blotting or reverse transcriptase–polymerase chain reaction (RT-PCR), techniques that assess protein or mRNA expression, respectively. Whether Ca_V_ channels are actually present in glioma cells remains unclear, and further studies are still required to verify the existence of physiologically functional Ca^2+^ currents. Therefore, these ion channels were not included in the scope of discussion in this review paper. Additionally, some transient receptor potential (TRP) channels may be expressed on the plasma membrane of glioma cells [113,114,115,116] and could influence the invasive behaviors of glioma; however, they were not included in the discussion in this article.

The docking studies presented in this study—based on structure-guided virtual screening—should be considered exploratory, offering qualitative perspectives on potential binding interactions rather than definitive structural conclusions. In the absence of experimental validation, these computational predictions must be interpreted with caution. Nevertheless, they provide valuable hypotheses that can inform subsequent experimental work, including cell-based assays, knockdown, and site-directed mutagenesis approaches [115,116]. Our findings suggest that the ion channel expressed in glioma may constitute a druggable target, characterized by a distinct docking site amenable to small-molecule modulation. Importantly, even subtle modifications in the atomic docking profiles of candidate compounds across different ion channels could considerably influence their ability to regulate the invasive or migratory behavior of neoplastic astrocytes.

It needs to be mentioned that, for ion channels, factors such as binding-site accessibility, conformational state, membrane environment, and ligand protonation may substantially influence docking results. The molecular docking shown here therefore has inherent limitations and should not be overinterpreted. These factors should be carefully considered when interpreting computational predictions. The docking analyses are presented as qualitative, hypothesis-generating predictions rather than definitive mechanistic evidence of ligand–channel interactions or functional modulation.

The proposed binding poses and residue interactions shown here represent computational models that require experimental validation, such as mutational analyses and new experimental studies. The reported docking scores and binding energies should not be interpreted as quantitative measures of binding affinity but rather as relative indicators for generating testable hypotheses. The complementary structural insights should be interpreted alongside the available experimental evidence.

It should also be clarified that molecular docking analyses were performed only for selected compounds rather than for all compounds included in this review. Docking studies were limited to representative compounds for which high-quality structural information and suitable protein models were available, allowing computational analyses to provide mechanistic insights that complement the existing experimental evidence. A systematic docking analysis of all compounds discussed in the review was beyond the scope of the present work because of limitations in structural availability, differences in ion channel conformational states, and the exploratory nature of the computational component.

In this study, modulation of ion channel activity alone does not establish a causal channel-mediated anti-glioma mechanism, particularly for pleiotropic compounds such as valproic acid, berberine, arecoline, triptolide, and oxaliplatin, which exert multiple biological effects through diverse molecular targets. However, their ion channel regulation could be considered to be a potential contributing mechanism that acts alongside other established molecular pathways [2,3]. Additional mechanistic studies are required to establish channel-mediated therapeutic effects [3].

By incorporating clinically approved agents and bioactive natural products, this study establishes a strong framework for drug-repurposing strategies. Leveraging these well-characterized compound classes therefore has the potential to significantly accelerate translational research.

**Figure 8:**
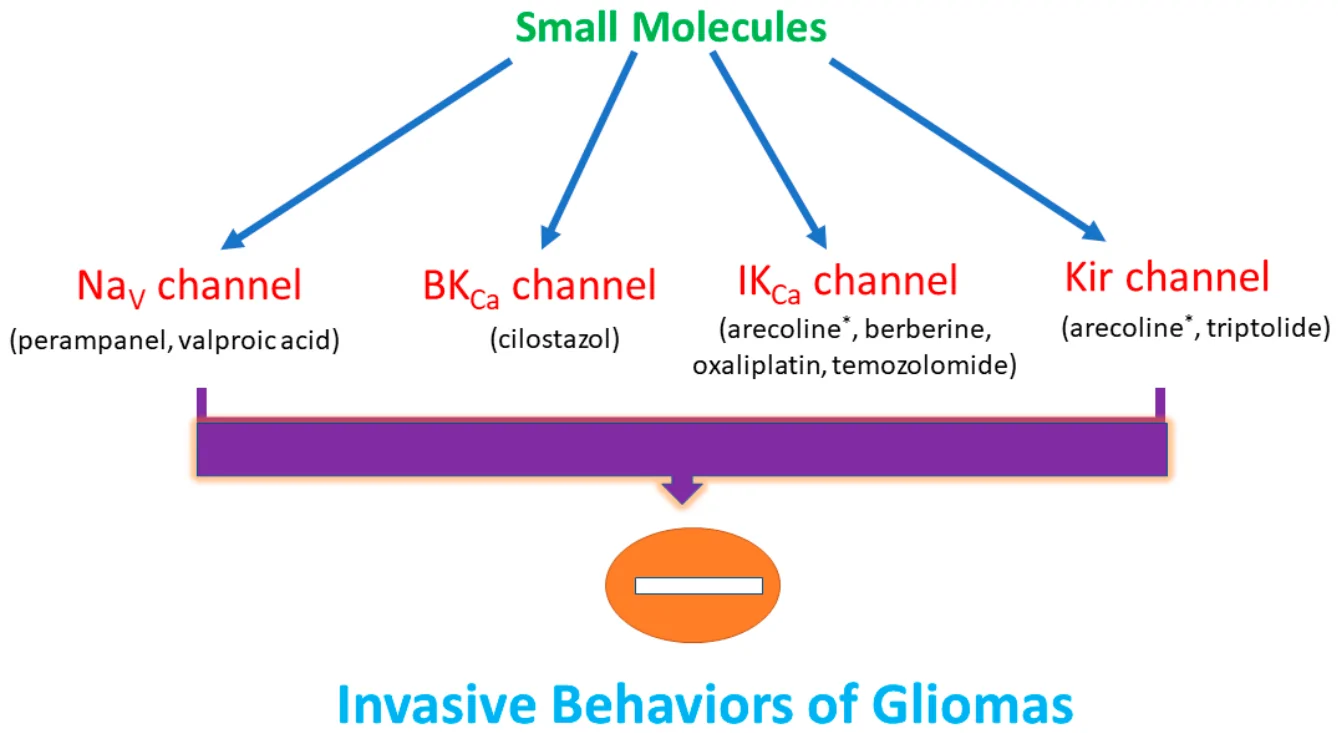
Proposed mechanisms by which small molecules suppress glioma invasiveness through modulation of ion channel activity. Schematic representation illustrating how numerous small molecules, drugs, natural compounds, and phytoconstituents discussed in this review inhibit the invasive behavior of glioma cells by targeting ion channels functionally expressed on the plasma membrane. These channels include Na_V_ channels, BK_Ca_ channels, IK_Ca_ channels, and Kir channels. The compounds shown in parentheses beneath each channel correspond to the agents described in the preceding sections of this article. The asterisk (*) indicates that arecoline has been reported to inhibit both IK_Ca_ and Kir channel activities in glioma cells. The highly invasive nature of gliomas contributes to several major clinical challenges [1,80,106,107], including: (1) the inability to achieve complete surgical resection; (2) resistance of infiltrating tumor cells to conventional therapies; (3) frequent tumor recurrence following treatment; and (4) limitations on surgical intervention imposed by the need to preserve critical neurological function. Targeting ion channel activity represents a compelling strategy to limit glioma invasion and improve therapeutic outcomes. The ion channel modulation described here should be considered a potential contributing factor or associative mechanism, rather than an established causal anti-glioma pathway.

Collectively, the findings of the present study suggest that numerous small-molecule compounds currently available through drug-repurposing strategies [3,8,9,26,30,60,78,108,113,116,117,118,119] are capable of modulating multiple ion channel types in glioma cells, including Na_V_ channels, BK_Ca_ channels, IK_Ca_ channels, and Kir channels. These compounds may influence channel gating properties, such as open probability and kinetic behavior, thereby attenuating the invasive and migratory capacities of glioma cells (Figure 8). However, it should be noted that these compounds, including arecoline, berberine, oxaliplatin, valproic acid, and triptolide, may exert neurotoxic effects through mechanisms other than ion channel interactions. Recent studies have applied artificial intelligence (AI) to investigate the relationship between glioma malignancy and the expression of ion channels [2,3,9,116,117,118,119]. Whether other functional voltage-gated ion channels are present on glioma cells of varying degrees of malignancy remains to be further investigated in future studies.

## Figures and Tables

**Table 1:** Pharmacological modulators targeting ion channels in glioma. The table summarizes the major classes of ion channels, representative pharmacological modulators, experimental concentrations, corresponding references, and molecular docking results. To facilitate interpretation, the evidence is categorized according to direct electrophysiological modulation, glioma-relevant phenotypic effects, in vivo or clinical evidence, causal evidence supporting ion channel involvement, and docking-based hypotheses. This classification highlights the different levels of experimental support and distinguishes computational predictions from experimentally validated findings.

Ion Channel Type	Modulator	IC_50_, EC_50_, or Concentration Range (μM) ^3^	Ref. ^5^	Docking Results ^6^
**Voltage-gated Na^+^ channel**	Perampanel	0.78 and 4.12 ^4^	[10,11]	(+) ^7^
	Valproic acid	30–100	[12]	
**Large-conductance Ca^2+^-activated K^+^ channel**	Cilostazol ^1^	1–30	[13,14]	(+)
**Intermediate-conductance Ca^2+^-activated K^+^ channel**	Arecoline ^2^	11.2 ^2^	[15]	(+)
	Berberine	50	[16]	
	Oxaliplatin	67	[17]	
	Temozolomide	9.2	[18]	
**Inwardly rectifying K^+^ channel**	Arecoline ^2^	1–30	[19]	(+)
	Triptolide	0.72	[20]	

^1^ Cilostazol acts by enhancing ion channel activity, whereas the other compounds have inhibitory effects on the amplitude of ionic currents. ^2^ Arecoline suppresses the activity of both intermediate-conductance Ca^2+^-activated K^+^ channels and inwardly rectifying K^+^ channels in glioma cells. The dissociation constant (K_D_) for arecoline-mediated inhibition of IK_Ca_ channels is 11.2 μM. ^3^ IC_50_ and EC_50_ represent the concentrations required to achieve half-maximal inhibition and half-maximal stimulation, respectively. In the absence of IC_50_ or EC_50_ values, the concentration range tested for each compound is presented as XX–XX μM. Unless otherwise indicated, all results were obtained using glioma cells. The effects of perampanel were evaluated in hippocampal mHippoE-14 neurons. ^4^ Perampanel suppresses the late and peak components of *I*_Na_ in hippocampal mHippoE-14 neurons at 0.78 and 4.12 μM, respectively. ^5^ Ref. refers to the bracketed reference number listed at the end of the manuscript. ^6^ (+) indicates that molecular docking results are presented in this paper. The detailed molecular docking procedures used in this study are provided in the Appendix A. ^7^ This study reports the molecular docking results of perampanel with both the Na_V_1.6 channel and the AMPA receptor.

## Data Availability

The original contributions presented in this study are included within the article. Further inquiries can be directed to the corresponding authors.

## Abbreviations

The following abbreviations are used in this manuscript:

AMPA receptor	α-Amino-3-hydroxy-5-methyl-4-isoxazolepropionic acid receptor
BK_Ca_ (KCNMA1/K_Ca_1.1) channel	Large-conductance Ca^2+^-activated K^+^ channel
Cil	Cilostazol
IK_Ca_ (KCNN4/K_Ca_3.1) channel	Intermediate-conductance Ca^2+^-activated K^+^ channel
*I*_K_	Voltage-gated K^+^ current
*I*_K(Ca)_	Ca^2+^-activated K^+^ current
*I*_Na_	Voltage-gated Na^+^ current
Kir channel	Inwardly rectifying K^+^ channel
Na_V_ (SCN) channel	Voltage-gated Na^+^ channel
TMZ	Temozolomide

## Appendix A

The three-dimensional (3D) conformers of the selected compounds were retrieved from the PubChem database (National Center for Biotechnology Information, National Library of Medicine, National Institutes of Health, Bethesda, MD, USA) in Structure Data File (SDF) format. The three-dimensional crystal structures of the target protein were obtained from the Research Collaboratory for Structural Bioinformatics Protein Data Bank (RCSB PDB). Here, “XXXX” is used generically to denote the corresponding PDB ID. The Protein Data Bank (PDB) is a global repository that archives experimentally determined three-dimensional structures of proteins, nucleic acids, and other biological macromolecules, providing essential structural information for molecular modeling and docking studies. In high-resolution X-ray protein structures such as 6V5A (BK_Ca_ or KCNMA1 channel), the remaining water molecules are those associated with the protein, whereas other PDB structures, such as 9DK5 (Na_V_1.6 or SCN8A channel), 9XJM (AMPA receptor), 9ZPT (IK_Ca_ or KCNN4 channel), and 8QQL (Kir2.1 or KCNJ2 channel), determined by electron microscopy, do not display any water atoms.

Molecular docking simulations were performed using PyRx version 0.8 (PyRx—Virtual Screening Tool; The Scripps Research Institute, La Jolla, CA, USA), utilizing the AutoDock Vina docking engine. The resulting docking poses were subsequently analyzed using BIOVIA Discovery Studio 2025 (Dassault Systèmes, San Diego, CA, USA) and LigPlot^+^ version 2.3 (European Bioinformatics Institute, Hinxton, Cambridge, UK) to visualize and characterize the protein–ligand interactions.

The overall workflow of the molecular docking study was as follows: PubChem (ligand retrieval) → Protein Data Bank (protein retrieval) → PyRx/AutoDock Vina (molecular docking) → BIOVIA Discovery Studio 2025 and LigPlot^+^ (protein–ligand interaction analysis)**.** The binding conformation with the lowest predicted binding energy (Mode 1) was selected for subsequent molecular interaction analyses [120,121].

The three-dimensional structures of the protein–ligand complexes and their molecular interactions were visualized and rendered using PyMOL version 3.1 (Schrödinger, LLC, New York, NY, USA). The corresponding PyMOL-generated images are presented in the left panels of each docking figure. The right panels of each docking figure illustrate the two-dimensional (2D) atomic interaction profiles of the protein–ligand complexes generated using LigPlot^+^ [109].
